# CXCL13 concentration in latent syphilis patients with treatment failure

**DOI:** 10.1515/med-2020-0204

**Published:** 2020-07-10

**Authors:** Yan Zhang, Jun Wang, Yingnan Wei, Huili Liu, Chunli Wu, Bin Qu, Yongxing Yan

**Affiliations:** Department of Neurology, The Third people’s Hospital of Hangzhou, Hangzhou, Zhejiang, China

**Keywords:** latent syphilis, neurosyphilis, CXCL13, cerebral spinal fluid

## Abstract

We aimed to investigate the CXCL13 concentration of the serum and cerebral spinal fluid (CSF) in human immunodeficiency virus (HIV)-negative latent syphilis patients with treatment failure and explore the change in CXCL13 after treatment. Sixty-eight latent syphilis patients with treatment failure (failure group), 68 syphilis patients with successful treatment (seroconversion group) and 18 patients with non-inflammatory diseases of the nervous system (control group) were included and serum and CSF were collected. Enzyme-linked immunosorbent assay was employed to detect the CXCL13 in the serum and CSF. Results showed that the serum CXCL13 concentration was comparable among three groups, and the CSF leukocyte count, IgG index and CXCL13 concentration in the failure group were significantly higher than those in the seroconversion group and control group (*P* < 0.05, *P* < 0.01). CSF CXCL13 concentration in the failure group was positively related to the CSF leukocyte count (*r* = 0.3594, *P* < 0.001). Of the 68 patients in the treatment failure group, neurosyphilis was found in 17 (25.0%). In conclusion, involvement of nervous system is one of the reasons for the treatment failure in patients with latent syphilis. Detection of CSF CXCL13 concentration is helpful for the diagnosis and therapeutic evaluation of HIV-negative latent syphilis patients with treatment failure and neurosyphilis.

## 1 Introduction

Syphilis is a sexually transmitted disease due to treponema pallidum infection and may cause damage to multiple important organs. Patients with latent syphilis may be asymptomatic for several years. In recent years, the annual increment in the incidence of syphilis is about 30% worldwide [1]. The epidemiological survey of infectious diseases sponsored by the Chinese Ministry of Health in February 2016 showed that syphilis has been the third most common infectious disease and the most common sexually transmitted disease with higher incidence than gonorrhea, and the prevalence of latent syphilis is also increasing [2,3]. Patients with latent syphilis have no or few symptoms, which may lead to the ignorance in these patients. However, the existing treponema pallidum of latent syphilis patients may persistently cause damage to tissues and organs, resulting in the development of neurosyphilis, which is more harmful than dominant syphilis. Several studies have reported the treatment failure in patients with latent syphilis [4,5,6]. Thus, clinicians should pay attention to the treatment failure in patients with latent syphilis and block its progression into neurosyphilis. Moreover, early diagnosis and early treatment are also crucial for the improvement of prognosis in these patients.

Previous studies have shown that the CXCL13 concentration of the cerebral spinal fluid (CSF) in human immunodeficiency virus (HIV)-negative/positive patients with neurosyphilis is significantly higher than in patients without neurosyphilis and thus CSF CXCL13 may serve as a marker in the diagnosis of neurosyphilis [7,8,9]. However, the relationship between CXCL13 concentration and latent syphilis is still poorly understood. In this retrospective study, the relationship of CXCL13 concentration of CSF and serum with latent syphilis was assessed in patients with treatment failure and the change in CXCL13 concentration of CSF and serum after standard treatment in these patients investigated.

## 2 Materials and methods

### 2.1 Subjects

Treatment failure group: a total of 68 latent syphilis patients due to treatment failure who received treatment from July 2011 to December 2012 in the Department of Dermatology and Sexually Transmitted Diseases were included in this study. There were 27 males and 41 females with the mean age of 39.6 ± 13.0 years (range: 19–72 years). Latent syphilis diagnosis criteria: (1) blood TRUST and treponema pallidum particle agglutination (TPPA) assay showed positive; patients were negative for HIV; (2) patients had no history of rash, the course of disease was unclear, and latent syphilis was diagnosed at blood donation, premarital examination, preoperative examination or routine examination during hospitalization; (3) latent syphilis at unknown stage was diagnosed, and serum TRUST titer was ≥1:4 at initial examination; and (4) patients were never diagnosed as having syphilis or received any anti-syphilis treatment. Latent syphilis with treatment failure diagnosis criteria: (1) latent syphilis patients received treatment with long-acting penicillin or procaine penicillin for at least one course, and the reduction of TRUST titer lower than four times or increase in TRUST titer was present after 6-month treatment; (2) re-infection and other nervous system diseases were excluded.

Control group: a total of 18 patients with neurological non-inflammatory disease who received treatment in our hospital in the same period were included. There were eight males and ten females with the mean age of 39.0 ± 13.4 years (range: 22–65 years). Eleven patients had migraine and seven were diagnosed with epilepsy.

Seroconversion group: a total of 68 syphilis patients with seroconversion were included. There were 36 males and 32 females with the mean age of 32.1 ± 9.8 years (range: 18–60 years). Primary syphilis was found in 32 patients, secondary syphilis in 24 and latent syphilis in 12. After anti-syphilis treatment, clinical symptoms and signs resolved, and seroconversion was confirmed by TRUST with the mean time of 14.2 ± 8.1 months.

### 2.2 Sample collection

Routine lumbar puncture was performed in all these patients, and 6 mL of CSF was collected for routine examination, biochemistry, detection of IgG index, TRUST, TPPA, cerebrospinal fluid-venereal disease research laboratory test and fluorescent treponemal antibody absorption test (FTA-ABS). At the same time, serum (5 mL) and CSF (2 mL) were harvested and stored at −80°C for further detection of CXCL13 concentration by enzyme-linked immunosorbent assay (ELISA). Lumber puncture, CXCL13 concentration in the serum and CSF were re-detected in patients diagnosed with neurosyphilis at 3 and 12 months after standard treatment.

### 2.3 Reagent and methods

TRUST kit (Shanghai Rongsheng Biotech Co., Ltd), TPPA kit (Japan Fuji Co., Ltd), VDRL kit (BD Company, USA), FTA-ABS kit (Trinity Biotech PLC, Ireland) and Human CXCL13/BLC/BCA-1 Quantikine ELISA Kit (RD company, Europe) were used in the present study. Detections were performed according to manufacturer’s instructions.

### 2.4 Treatments

Lumbar puncture was performed in all the patients. According to the diagnostic criteria for neurosyphilis [5], patients with neurosyphilis were treated with intravenous penicillin sodium (2,40,00,000 U/d; 40,00,000 per hour) (North China Pharmaceutical Company, China, Batch number: F1109206) for consecutive 14 days and intramuscular benzathine penicillin G (24,00,000 U; once weekly) (North China Pharmaceutical Company, China, Batch number: P1110147) for consecutive 3 weeks. Patients without neurosyphilis were intramuscularly treated with benzathine penicillin G (24,00,000 U/d; once weekly) (North China Pharmaceutical Company, China, Batch number: P1110147) for consecutive 3 weeks.

### 2.5 Ethics

All procedures involving human subjects were conducted in accordance with the ethics standards and with the Declaration of Helsinki as revised in 1983. And this study has been approved by the Ethics Committee of our hospital (No. 2011A018).

### 2.6 Statistical analysis

Statistical analysis was performed with SPSS version 20.0. Qualitative data were compared by the Chi square test. Quantitative data were subjected to normal distribution test with the Kolmogorov–Smirnov method. Quantitative data with normal distribution are expressed as mean ± standard deviation (\bar{x}\pm \text{SD}). Multigroup comparisons of the means were carried out by one-way analysis of variance test with post hoc analysis by the Student–Newman–Keuls test. Quantitative data with abnormal distribution are expressed as median and interquartile range (M[QR]) and compared with Kruskal–Wallis *H* test among groups or Mann–Whitney *U* test between groups. Correlation was evaluated with Spearman correlation analysis. A value of two-tailed *P* < 0.05 was considered statistically significant. Using receiver operating characteristic (ROC) analysis to evaluate the diagnostic value of CXCL13 concentration in the treatment failure latent syphilis, and using the multivariate logistic regression analysis to calculate the Youden’s index to determine the cut-off value, *P* < 0.05 was statistically significant.

## 3 Results

### 3.1 Patients’ characteristics at baseline

At baseline, there were no marked differences in age and gender characteristics among three groups (*F* = 2.4195, *F* = 2.6950, *P* > 0.05). The initial serum TRUST titer ≤1:32 at baseline was comparable between the treatment failure group and seroconversion group (*P* > 0.05). While the proportion of patients with serum TRUST titer ≥1:64 in treatment failure group was higher than in seroconversion group (*P* < 0.05; Table 1).

**Table 1:** Patients’ characteristics at baseline in three groups

	Treatment failure group (*n* = 68)	Seroconversion group (*n* = 68)	Control group (*n* = 18)	*F*/*X* ^2^	*P*
Gender (M/F) (*n*)	27/41	36/32	8/10	2.4195	0.2983
Age (year)	39.6 ± 13.0	32.1 ± 9.8	39.0 ± 13.4	2.6950	0.0712
Blood TRUST titer (*n*)					
1:4	7	5	0	0.3656	0.5454
1:8	8	6	0	0.3185	0.5725
1:16	23	32	0	2.4727	0.1158
1:32	17	21	0	0.6663	0.4143
≥1:64	13	4	0	4.3025	0.0380

Of the 68 patients in the treatment failure group, neurosyphilis was found in 17 patients (25.0%, 17/68). There were 11 males and 6 females with the age range of 31–66 years (include symptomatic neurosyphilis that was noted in 9 patients and asymptomatic neurosyphilis in 8). Non-neurosyphilis was noted in 51 patients, there were 16 males and 35 females with the mean age of 37.2 ± 12.8 years (range: 18–72 years), compared with non-neurosyphilis, where the majority of neurosyphilis were male, and the difference was statistically significant (*P* < 0.05). In these neurosyphilis patients, the initial serum TRUST titer ≥1:16 was found in 16 patients at baseline, while only one patient had a serum TRUST titer <1:16. Patients diagnosed as having neurosyphilis with serum TRUST titer ≥1:16 were 4.5 times more than those with serum TRUST titer <1:16 (30.2% vs 6.7%; 16/53 vs 1/15), although significant difference was not observed (*P* > 0.05; Table 2).

**Table 2:** Sixty-eight patients’ characteristics in the treatment failure group

	Symptomatic neurosyphilis (*n* = 9)	Asymptomatic neurosyphilis (*n* = 8)	Non-neurosyphilis (*n* = 51)	*F/X* ^*2*^	*P*
Gender (M/F) (*n*)	7/2	4/4	16/35	7.2825	0.0262
Age (years)	47.8 ± 9.3	45.5 ± 13.3	37.2 ± 12.8	1.2550	0.5338
Blood TRUST titer (*n*)					
<1:16	0	1	14	NS	NS
≥1:16	9	7	37	3.8348	0.1470

NS: not significant.

### 3.2 CSF parameters in three groups

There was no significant difference in the intracranial pressure among three groups (*F* = 0.1445, *P* > 0.05). In the treatment failure group, the CSF leukocyte count and IgG index were significantly higher than in the seroconversion group and the control group (*H* = 6.868, *P* < 0.05 and *F* = 9.559, *P* < 0.01, respectively), and the CSF protein content was slightly higher than in the seroconversion group and the control group, but the difference was not significant (*F* = 1.136, *P* > 0.05). No significant differences were observed between the seroconversion group and the control group (*P* > 0.05; Table 3).

**Table 3:** CSF and serum CXCL13 concentration in three groups

	Intracranial pressure (mmH_2_O)	CSF protein (mg/dL)	CSF leukocyte (×10^6^/L)	CSF IgG index	CXCL13 concentration (pg/mL)	
					Blood	CSF
Treatment failure (*n* = 68)	141.6 ± 24.3	36.9 ± 51.0	2.5 (7.75)	0.86 ± 0.55	112.4 (73.2)	16.85 (46.807)
Non-neurosyphilis (*n* = 51)	140.78 ± 24.8	33.0 ± 57.3	2.0 (4.0)	0.63 ± 0.30	106.1 (70.21)	10.01 (15.38)
Neurosyphilis (*n* = 17)						
Baseline	141.2 ± 24.2	48.4 ± 20.8	21 (21.0)^☆^	1.54 ± 0.68^☆^	147.9 (82.79)	124.4 (111.57)^☆^
3 months	137.6 ± 16.4	44.1 ± 19.0	8 (16.0)^▲^	1.0 ± 0.5^▲^	141.6 (78.11)	11.45 (13.53)^▲▲^
12 months	148.2 ± 21.4	39.5 ± 14.1	5 (8.0)^▲▲##^	0.58 ± 0.20^▲▲#^	140.7 (78.37)	4.51 (5.875)^▲▲#^
Seroconversion (*n* = 68)	143.1 ± 19.87	28.56 ± 13.26	2 (4.0)*	0.58 ± 0.19**	126.2 (64.65)	4.7 (3.63)**
Control (*n* = 18)	140.28 ± 22.0	27.19 ± 11.89	2 (1.5)*	0.56 ± 0.21**	110.5 (40.74)	4.805 (4.005)**
*F*/*H* value	0.1445	1.136	6.868	9.559	0.0059	66.82
*P* value	0.8656	0.3237	0.0323	0.0001	0.9971	0.0000

*P < 0.05 vs treatment failure group; **P < 0.01 vs treatment failure group; ^☆^ *P* < 0.01 vs non-neurosyphilis group; ^▲^ *P* < 0.05 vs baseline; ^▲▲^ *P* < 0.01 vs baseline; ^#^ *P* < 0.05 vs 3 months; and ^##^ *P* < 0.01 vs 3 months.

### 3.3 Serum and CSF CXCL13 concentrations in three groups

**Figure 1:**
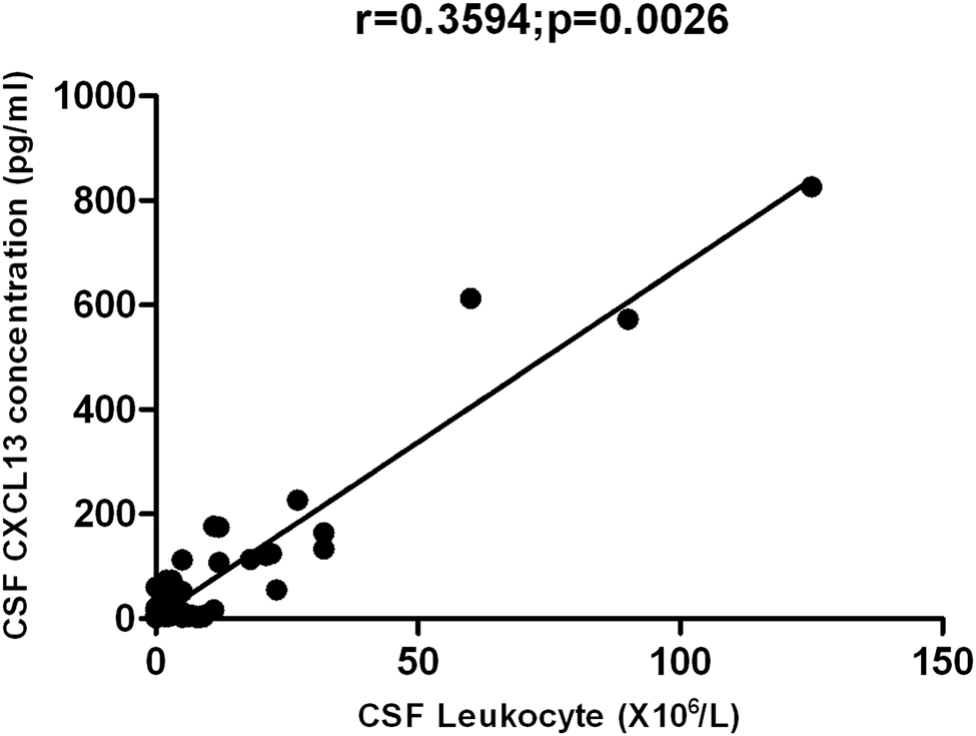
The leukocyte count in CSF was positively related to the CSF CXCL13 concentration (*r* = 0.3594, *p* = 0.0026).

There was no marked difference in the serum CXCL13 among three groups (*H* = 0.0059, *P* > 0.05). The CSF CXCL13 concentration in the treatment failure group was significantly higher than in the seroconversion group and the control group (*H* = 66.82, *P* < 0.01), but there was no marked difference between the seroconversion group and the control group (*P* > 0.05; Table 3). The CSF leukocyte count had a positive relationship with CSF CXCL13 concentration in the treatment failure group (*r* = 0.3594, *P* = 0.0026; Figure 1). But there was no significant correlation between serum and CSF CXCL13 concentrations in different groups (*P* > 0.05).

### 3.4 ROC curve analysis

**Figure 2:**
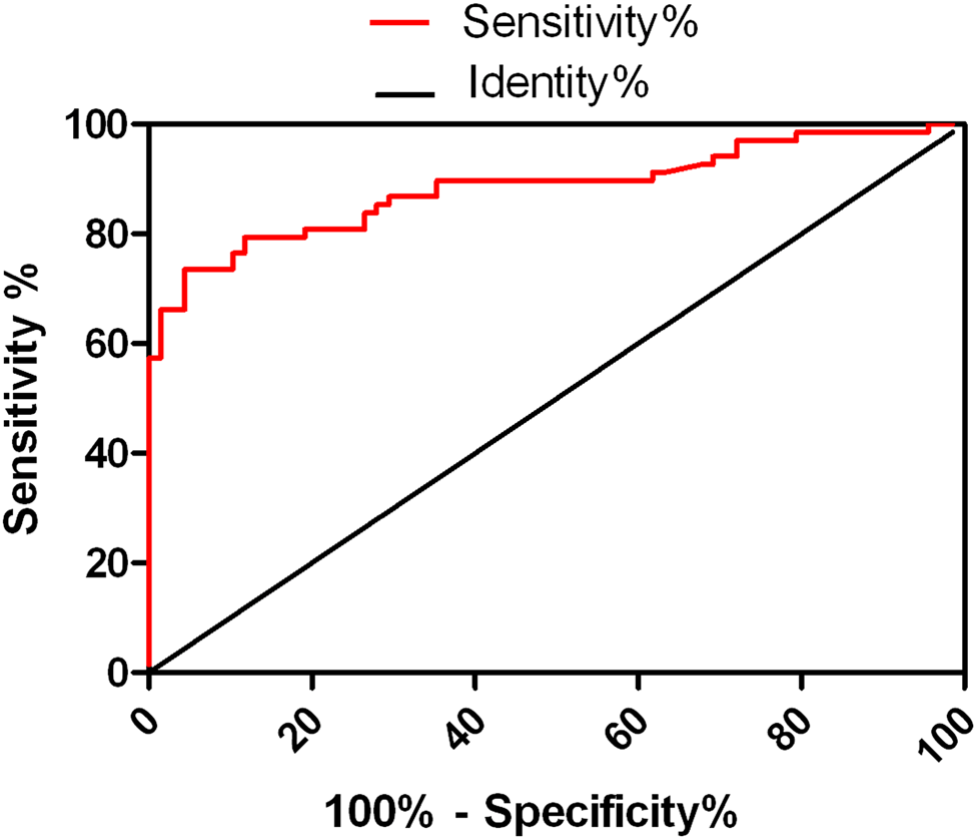
The area under curve of CXCL13 concentration in CSF to diagnosis the treatment failure of latent syphilis.

As per the area under curve of CXCL13 concentration in CSF to diagnosis the treatment failure of latent syphilis was 0.8838 (95% CI: 0.8245–0.9430, *P* < 0.01). When the cutoff value was 9.41 pg/mL, the sensitivity and the specificity were 73.53% and 95.59%, respectively (Figure 2).

### 3.5 Multivariate binary logistic regression analysis

Taking treatment failure of the latent syphilis as the dependent variable, the variables in Tables 1 and 3 with *P* < 0.1 are independent variables (age; initial serum TRUST titer ≥1:64; CSF leukocyte count; CSF IgG index; CSF CXCL13 concentration). Multivariate binary logistic regression analysis shows that the concentration of CXCL13 in CSF and the initial serum TRUST titer ≥1:64 are the risk factors for predicting the treatment failure of latent syphilis (Table 4).

**Table 4:** Multivariate binary logistic regression analysis

Variable	*β* value	SE value	Wald	OR value	95% CI	*P* value
Age	0.035	0.024	2.031	1.035	0.987–1.086	0.154
Initial serum TRUST titer ≥1:64	−2.266	0.948	5.711	0.104	0.016–0.665	0.017
CSF leukocyte (×10^6^/L)	0.075	0.091	0.676	1.078	0.902–1.288	0.411
CSF IgG index	1.773	1.282	1.913	5.890	0.477–72.669	0.167
CSF CXCL13 concentration (pg/mL)	0.480	0.102	21.948	1.616	1.322–1.975	0.000

### 3.6 CSF parameters and serum/CSF CXCL13 concentration in 17 patients with neurosyphilis

**Figure 3:**
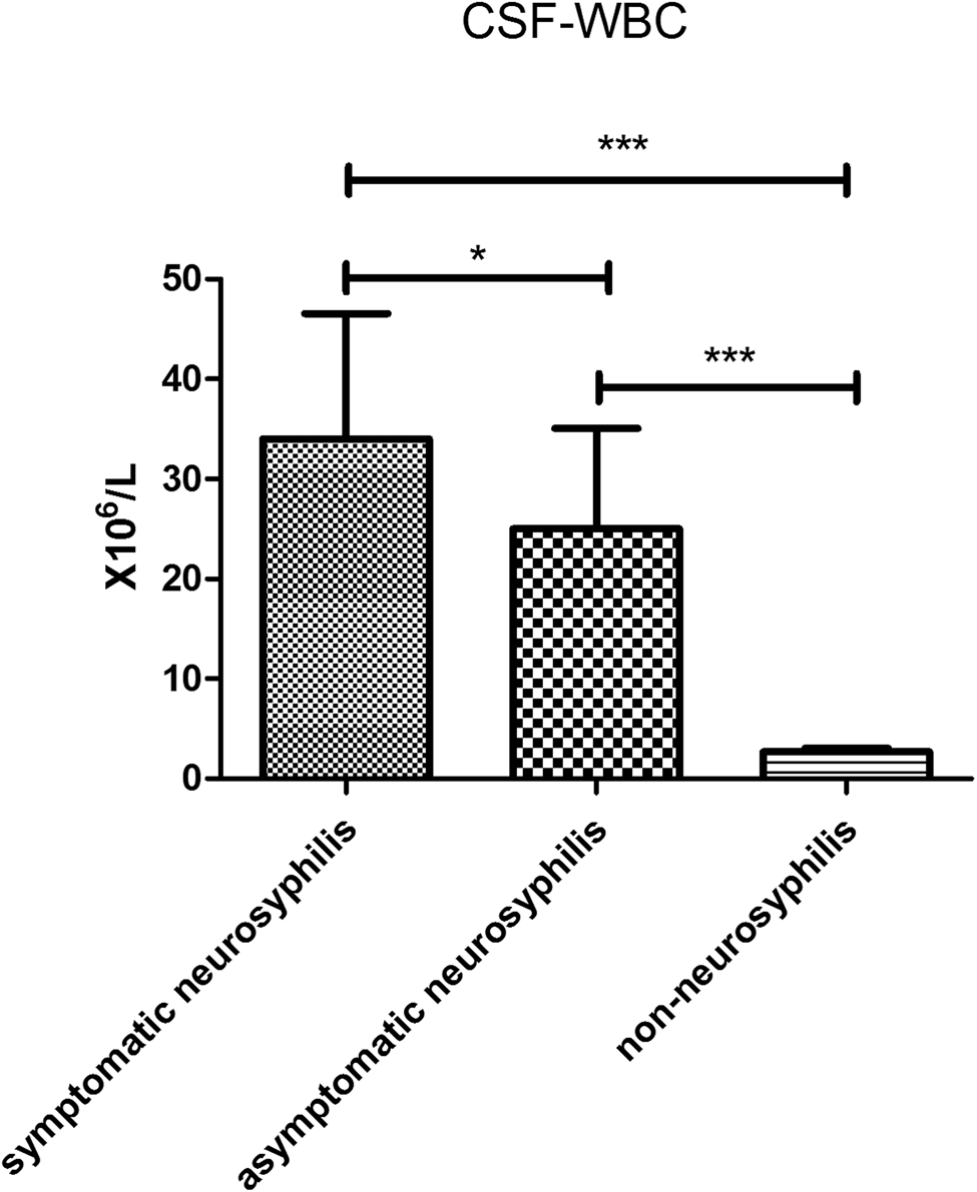
The comparison in WBC count among three groups. *p < 0.05; ***p < 0.01.

**Figure 4:**
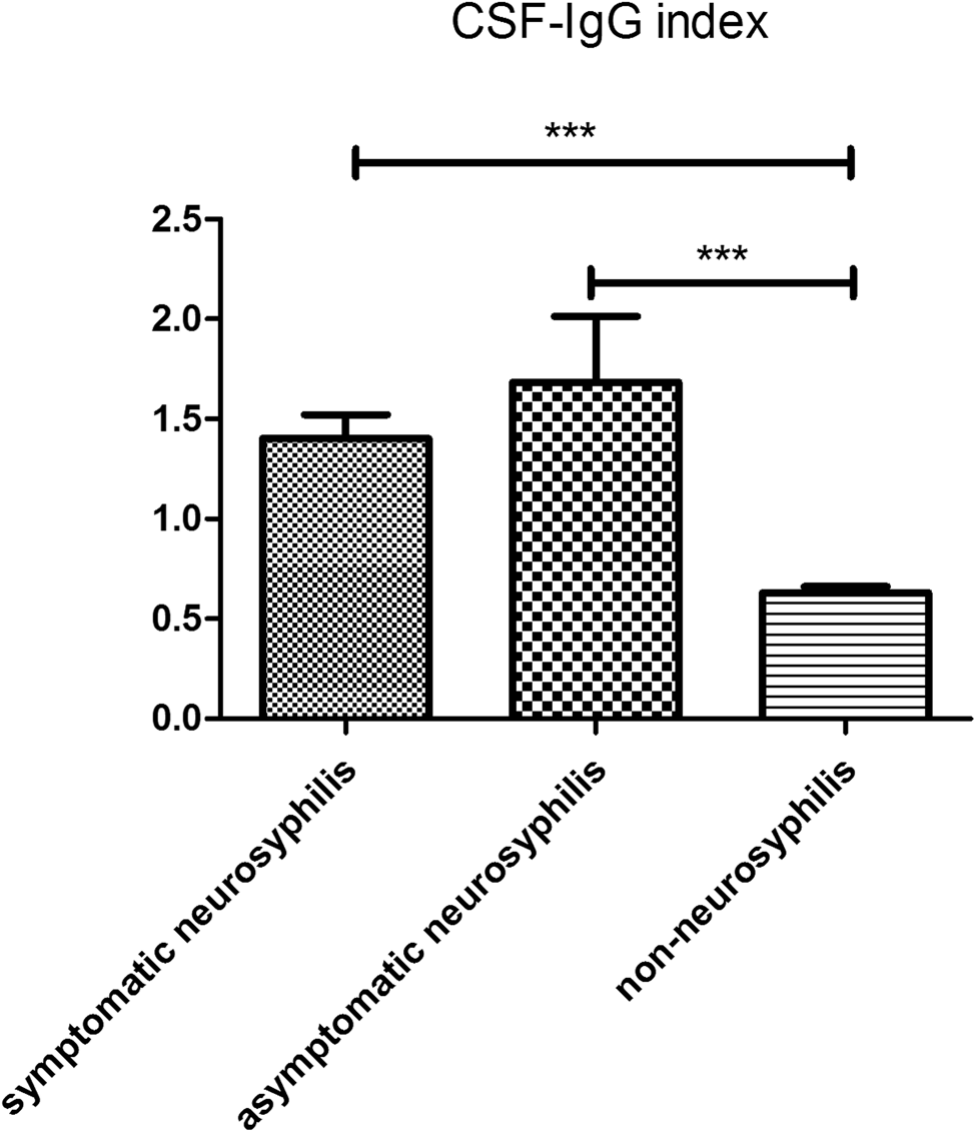
The comparison in IgG index among three groups. ***p < 0.01.

**Figure 5:**
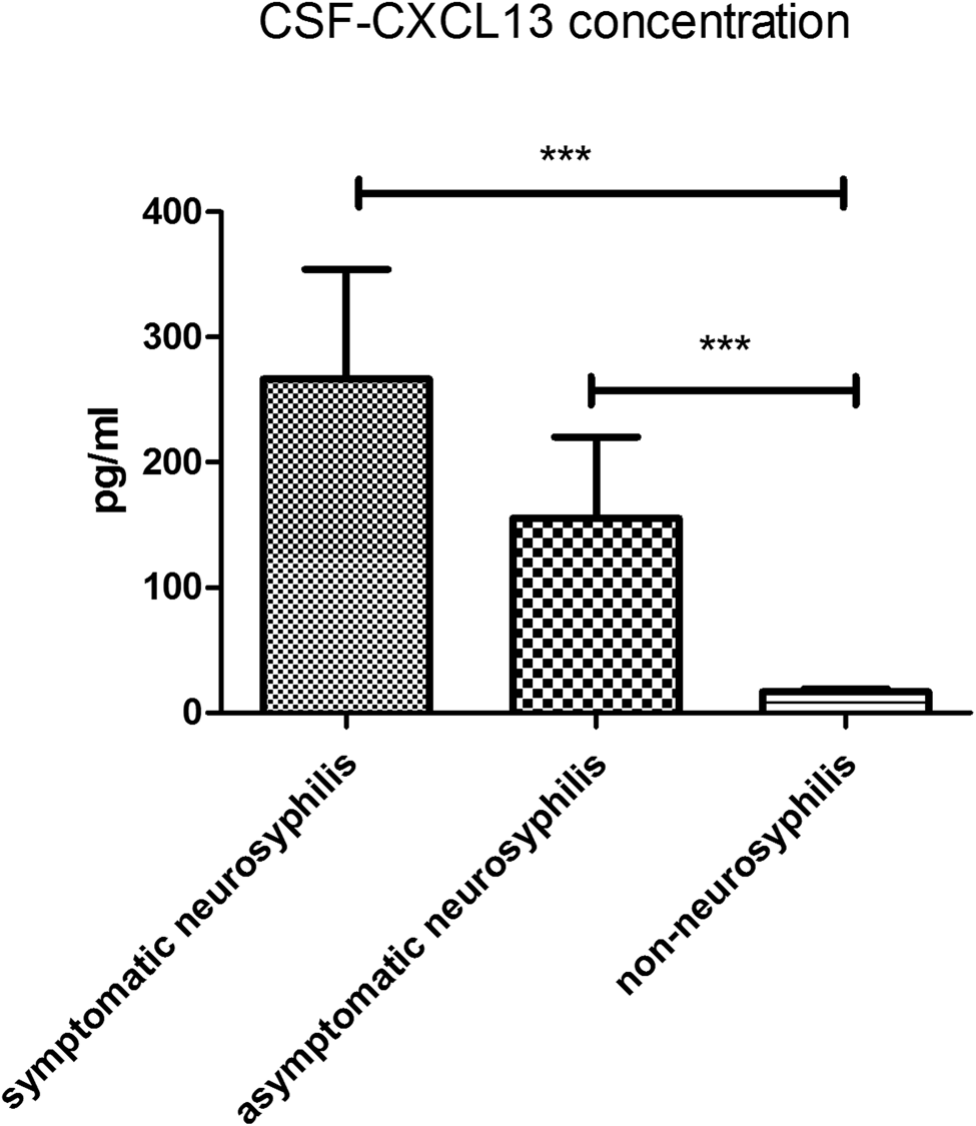
CXCL13 concentration in CSF among three groups. ***p < 0.01.

In symptomatic neurosyphilis patients (*n* = 9), the CSF leukocyte count was 21 × 10^6^/L, which was markedly higher than in asymptomatic neurosyphilis patients (*n* = 8) (17.5 × 10^6^/L; *P* < 0.05) and non-neurosyphilis patients (*n* = 51) (2 × 10^6^/L; *P* < 0.01). In addition, the CSF leukocyte count in asymptomatic neurosyphilis patients was significantly higher than in non-neurosyphilis patients (*P* < 0.01; Figure 3). The IgG index in asymptomatic (1.40 ± 0.35) and symptomatic (1.68 ± 0.93) neurosyphilis patients was significantly higher than in non-neurosyphilis patients (0.63 ± 0.30; *P* < 0.01; Figure 4). In symptomatic and asymptomatic neurosyphilis patients, the CSF CXCL13 concentrations were 133.5 pg/mL and 90.63 pg/mL, respectively, which were significantly higher than in non-neurosyphilis patients (10.01 pg/mL; *P* < 0.01; Figure 5).

In neurosyphilis patients (*n* = 17), after 3-month treatment, the CSF leukocyte count, IgG index and CXCL13 concentration reduced significantly as compared to those before treatment; they further reduced after 12-month treatment. The CSF leukocyte count, IgG index and CXCL13 concentration after 12-month treatment were markedly lower than those before treatment and after 3-month treatment (*P* < 0.05 or *P* < 0.01; Table 3). The CSF leukocyte count had a positive relationship with CSF CXCL13 concentration in 17 patients with neurosyphilis (*r* = 0.8855, *p* < 0.01). However, there was no correlation between serum and CSF CXCL13 concentrations in patients with neurosyphilis (*P* > 0.05).

## 4 Discussion

Syphilis is a kind of systemic and chronic disease, which can invade almost all organs and produce a variety of symptoms and signs. Once the nervous system is involved, there will be early functional damage or late irreversible organic abnormality, even life-threatening. Syphilis can also be in a long-term latent state; latent syphilis refers to a disease without clinical symptoms and positive result in the syphilis serum test after exclusion of diseases causing positive results in the syphilis serum test. It affects the physical and mental health of patients seriously, so early diagnosis and treatment of the disease are very important.

In this study, lumbar puncture and subsequent examination confirmed neurosyphilis in 17 patients in 68 latent syphilis patients due to treatment failure, with the prevalence of 25% (17/68). Syphilis at any stage has the possibility to cause damage to the central nervous system. Thus, CSF examination is recommended for syphilis patients with central nervous system involvement or treatment failure after standard anti-syphilis therapy. However, lumbar puncture and subsequent CSF examination are seldom employed in clinical practice. Thurnheer et al. [10] found that only 35% of patients received CSF examination according to the recommendation of clinicians. Agmon-Levin et al. [11] investigated 150 HIV positive patients with syphilis, who received treatment in the Israel AIDS Center between 2000 and 2005, and found that only 51 patients (34%) received CSF examination, in whom 16 (31%) had abnormal results in CSF examination and were diagnosed with neurosyphilis. In this study, among 68 latent syphilis patients with treatment failure receiving lumbar puncture for screening, there were 27 males and 41 females, suggesting that more females are willing to receive lumbar puncture, which may be ascribed to the improved compliance of females due to the requirement for fertility. Of note, in 17 patients with neurosyphilis, there were 11 males and 6 females, suggesting gender predisposition. Thus, medical education should be improved in male patients and clinicians should encourage male patients to receive timely lumbar puncture for screening.

After treponema pallidum infection, the treponema pallidum induced immune reaction plays important roles in the occurrence, development and cure of syphilis [12]. Marra et al. [13] found that the level of B cells in cerebrospinal fluid of neurosyphilis patients with HIV infection increased significantly, which was mainly related to the infection of neurosyphilis, but not related to HIV infection. There is evidence showing that cytokines produced after treponema pallidum infection serve as important mediators in the immune response and are involved in the subsequent pathology [14,15,16]. Chemokines are also a group of cytokines secreted by multiple cell types. Chemokine (C-X-C motif) ligand 13 (CXCL13) is a member of CXC chemokine family and was first reported by Legler et al. [17] in 1998. It is mainly secreted by secondary lymphoid tissue, lymph nodes and dendritic cells and has the ability to attract B cells, also known as B-lymphocyte chemokine (BLC). It regulates the settlement and movement of B cells and follicular CD4+ T-helper cells through its homologous receptor CXCR5 and plays important roles in the migration and homing of lymphocytes in CSF. It is considered to be the main factor of recruiting B cells in the neuroinflammatory response and participates in various immune inflammatory responses in the related central nervous system diseases, such as multiple sclerosis, neurological Lyme disease, anti-NMDA receptor encephalitis, HIV encephalopathy and neurosyphilis. It may be used as a biomarker for diagnosis of these diseases [18,19,20,21,22].

This study also found that the CXCL13 concentration of CSF in latent syphilis patients with failure treatment was significantly higher than that in the seroconversion group (*P* < 0.01), and the CSF leukocyte count was positively correlated with the concentration of CXCL13; we also found that there was no significant difference in serum CXCL13 concentration between the failure treatment group and the seroconversion group and control group. The concentration of CXCL13 in CSF in the failure treatment group was significantly higher than those of the other two groups. These findings indicate that the increased CXCL13 in the CSF of latent syphilis patients with failure treatment may be synthesized by cells after the stimulation of treponema pallidum in the nervous system, but not from the peripheral blood. This suggests that the concentration of CSF CXCL13 has certain reference value for the treatment efficacy of latent syphilis with failure treatment. The ROC curve analysis found that when the concentration of CXCL13 in CSF was 9.41 pg/mL, it had the greatest value in predicting whether there was any treatment failure of the latent syphilis, the sensitivity and specificity were 73.53% and 95.59%, respectively. At the same time, multivariate regression analysis showed that the concentration of CXCL13 in CSF and the initial serum TRUST titer ≥1:64 are the risk factors for the treatment failure of latent syphilis and may be used as markers to predict whether there was any treatment failure of the latent syphilis.

When latent syphilis patients have obvious neurological symptoms or signs, they are easy to undergo lumbar puncture, but many latent syphilis patients have no obvious symptoms. Performing lumbar puncture to determine whether the central system infection in all patients is obviously unrealistic, so it needs an effective warning marker to determine whether to perform the lumbar puncture examination for latent syphilis patients with treatment failure. Choe et al. [23] performed lumbar puncture in 70 untreated latent syphilis patients with HIV negative, the serum RPR titer was <1:16 in all the patients and neurosyphilis accounted for 6.2%. In our study, the detection rate of neurosyphilis was 6.7% (1/15) in latent syphilis patients with failure treatment whose initial TRUST titer <1:16, similar to the Choe et al. study. But in patients with an initial TRUST titer ≥1:16, the incidence of neurosyphilis was higher (30.2% (16/53) vs 6.7% (1/15)), and the detection rate of neurosyphilis in our study was higher than that reported by Choe et al. This might be ascribed to high serum TRUST titer at baseline in these patients. At the same time, after multivariate regression analysis we found that the initial serum TRUST titer ≥1:64 is one of the risk factors for treatment failure of the latent syphilis. So, we speculate that the incidence of neurosyphilis in latent syphilis patients with failure treatment is related to the initial serum TRUST titer. Nervous system involvement may be one of the reasons for treatment failure in latent syphilis patients. For latent syphilis patients who have serum TRUST titer ≥1:64, it is important to perform lumbar puncture for screening in time and it can reduce the missed diagnosis and misdiagnosis rate of neurosyphilis.

Intravenous injection of high-dose penicillin is a treatment of choice for neurosyphilis. In this study, among the 68 cases of latent syphilis with failure treatment, 17 cases of neurosyphilis were found. After 2 weeks of standard treatment with high-dose penicillin, CSF leukocyte count, protein concentration and IgG index were detected during follow-up at 3 and 12 months. Results showed that these parameters reduced significantly and tended to become normal after treatment. This suggests that treatment is effective. In addition, the CSF CXCL13 concentration reduced dramatically after treatment, similar to the previous research results of Yan et al. [24] and Hu et al. [7]. Thus, it is suggested that CSF CXCL13 concentration may be helpful for the diagnosis and therapeutic evaluation of neurosyphilis. We speculate that the level of CXCL13 in CSF may be a marker to detecting whether latent syphilis patients are centrally infected and monitoring the treatment effect.

There were limitations in this study. The sample size was small and the follow-up was relatively short. In addition, whether CSF CXCL13 is more sensitive than leukocyte count is required to be validated in more patients with syphilis at different stages. Anyway, our findings provide evidence for the treatment of latent syphilis.

## 5 Conclusion

Central nervous system involvement is one of the reasons for treatment failure in latent syphilis patients, lumbar puncture for screening timely can reduce the missed diagnosis rate of neurosyphilis, and CSF CXCL13 concentration is helpful for the diagnosis and evaluation of therapeutic efficacy of latent syphilis patients with treatment failure and neurosyphilis. However, more controlled studies are required to elucidate the change in CXCL13 after treatment in patients with syphilis at different stages.
